# The structure of the ternary Eg5–ADP–ispinesib complex

**DOI:** 10.1107/S0907444912027965

**Published:** 2012-09-13

**Authors:** S. K. Talapatra, A. W. Schüttelkopf, F. Kozielski

**Affiliations:** a Molecular Motor Laboratory, The Beatson Institute for Cancer Research, Garscube Estate, Switchback Road, Bearsden, Glasgow G61 1BD, Scotland, UK

**Keywords:** Eg5, kinesins, ispinesib

## Abstract

The human kinesin Eg5 is responsible for bipolar spindle formation during early mitosis. Inhibition of Eg5 triggers the formation of monoastral spindles, leading to mitotic arrest that eventually causes apoptosis. There is increasing evidence that Eg5 constitutes a potential drug target for the development of cancer chemotherapeutics. The most advanced Eg5-targeting agent is ispinesib, which exhibits potent antitumour activity and is currently in multiple phase II clinical trials. In this study, the crystal structure of the Eg5 motor domain in complex with ispinesib, supported by kinetic and thermodynamic binding data, is reported. Ispinesib occupies the same induced-fit pocket in Eg5 as other allosteric inhibitors, making extensive hydrophobic interactions with the protein. The data for the Eg5–ADP–ispinesib complex suffered from pseudo-mero­hedral twinning and revealed translational noncrystallographic symmetry, leading to challenges in data processing, space-group assignment and structure solution as well as in refinement. These complications may explain the lack of available structural information for this important agent and its analogues. The present structure represents the best interpretation of these data based on extensive data-reduction, structure-solution and refinement trials.

## Abstract 2

The complex between the motor protein Eg5 and the phase II clinical candidate ispinesib provides insights into the mechanism of action of this important class of inhibitors.

## 1. Introduction   

Proteins involved in mitosis are attractive potential targets for cancer therapy, as their inhibition may allow the specific targetting of proliferating cells (Bergnes *et al.*, 2005 ▶; Harrison *et al.*, 2009 ▶). Indeed, there are a number of antimitotic drugs in clinical use, all of which target microtubule (MT) dynamics. Unfortunately, however, these drugs show significant side effects, as MTs are involved in a wide variety of cellular processes aside from mitosis.

Kinesin motor proteins move along MTs in an ATP-dependent manner. While they are conventionally thought to function in cellular cargo transport, a number of kinesins have been found to act during mitosis (Wordeman, 2010 ▶), making them potential targets for antimitotic drugs (Good *et al.*, 2011 ▶). In fact, some of the mitotic kinesins appear to function exclusively during mitosis and as such they may deliver on the promise of an improved side-effect profile in anticancer therapy through inhibition of mitotic proteins.

Human Eg5 (KSP, kinesin spindle protein, KIF11), a member of the kinesin-5 family (Miki *et al.*, 2003 ▶), is a well characterized mitotic kinesin that is required to establish a bipolar mitotic spindle. Eg5 forms homotetramers that can attach to neighbouring antiparallel spindle MTs and slide them against each other, thus separating the duplicated centrosomes (Kapitein *et al.*, 2005 ▶).

Loss of Eg5 function owing to RNA interference or small-molecule inhibitors results in the formation of monoastral spindles, cell-cycle arrest and apoptosis (Blangy *et al.*, 1995 ▶; Weil *et al.*, 2002 ▶; Mayer *et al.*, 1999 ▶). A number of small-molecule inhibitors of Eg5 have been identified, including MK-0731, pyrrolotriazin-4-one-based inhibitors and the quinazolin-4-­one-based ispinesib (Cox & Garbaccio, 2010 ▶; Lad *et al.*, 2008 ▶; Kim *et al.*, 2006 ▶), all of which are allosteric inhibitors that bind to the unique L5 loop region of the catalytic domain. Ispinesib (also named SB-715992 or CK0238273) is a potent and selective inhibitor of Eg5 that is currently in multiple phase II clinical trials (Burris *et al.*, 2011 ▶; Souid *et al.*, 2010 ▶) and is one of the most advanced drug candidates. The importance of the quinazolin-4-one scaffold targeting Eg5 is further underlined by the fact that three structurally related compounds are in various stages of clinical development: SB-­743921, a second-generation ispinesib analogue (Holen *et al.*, 2011 ▶), AZD4877 (Esaki *et al.*, 2011 ▶) and Arq621 (Chen *et al.*, 2011 ▶). A greater understanding of the molecular details of the protein–inhibitor interactions of this class of compounds is therefore crucial.

Although an Eg5–ispinesib complex has been reported previously (Zhang *et al.*, 2008 ▶), no coordinates or experimental data were made available, which hampers detailed analysis of this important enzyme–inhibitor inter­action and the use of the complex for further structure-guided design. Here, we report the 2.6 Å resolution structure of the ternary complex of the Eg5 motor domain in complex with Mg^2+^ADP and ispinesib. The structure provides a detailed overview of the interaction between ispinesib and the Eg5 motor domain and a rationale for further drug development.

## 2. Methods   

### 2.1. Cloning, expression and purification of Eg5   

The motor domain of human Eg5 (residues 1–368) was cloned, expressed and purified as described previously (Kaan *et al.*, 2010 ▶).

### 2.2. Steady-state ATPase-activity assay   

Steady-state basal and MT-stimulated ATPase rates were measured using the pyruvate kinase/lactate dehydrogenase linked assay (Hackney & Jiang, 2001 ▶). The amounts of Eg5 were optimized to 80–100 n*M* for basal and 5 n*M* for MT-stimulated activity assays. The IC_50_ values for the inhibition of the basal and MT-stimulated ATPase activities of Eg5 were measured for ispinesib up to 3.0 and 1.5 µ*M*. The ATP concentration was fixed at 1 m*M* and MTs were used at 2 µ*M* where applicable. Data were analysed using *Kaleidagraph* v.4.0 (Synergy Software). ATPase measurements were performed at 298 K using a 96-well Sunrise photometer (Tecan, Mannesdorf, Switzerland). MTs were prepared from lyophilized tubulin (tebu-bio catalogue No. 027T240-B) as described previously (Kozielski *et al.*, 2007 ▶).

### 2.3. Isothermal titration microcalorimetry (ITC)   

ITC was performed as described previously by Sheth *et al.* (2009 ▶) with minor modifications. Purified Eg5 was subjected to gel-filtration chromatography in buffer *A* (20 m*M* PIPES pH 6.8, 300 m*M* NaCl, 2 m*M* β-mercaptoethanol) to remove excess ATP and was then dialyzed overnight against buffer *A* supplemented with 0.5 m*M* ADP and 5 m*M* MgCl_2_. The protein was diluted to a final concentration of 20 µ*M* with dialysis buffer. The protein concentration was then verified by absorption measurements at 280 nm employing an experimental extinction coefficient determined using Eg5 denatured in 6.7 *M* guanidine hydrochloride with 20 m*M* phosphate pH 7.0 and including the absorption of ADP. Finally, 1% DMSO was added to the protein solution. The inhibitor was prepared in 100% DMSO and then diluted in dialysis buffer to a final concentration of 250 µ*M* ispinesib with 1% DMSO. All solutions were centrifuged for 5–10 min at room temperature prior to loading of the samples into the ITC cell. ITC experiments were performed with a Microcal VP-ITC titration calorimeter (Microcal Inc., North Hampton, Massachusetts, USA). All titrations were carried out at 298 K with a stirring speed of 350 rev min^−1^. A total of 26 injections were performed per titration; the first injection of 5 µl was followed by 25 injections of 10 µl with a gap of 240 s between them. The heat of dilution was subtracted prior to data analysis. The thermodynamic parameters *n* (stoichiometry), *K*_a_ (association constant) and Δ*H* (enthalpy change) were obtained through fitting of the experimental data using the single-site binding model of the *Origin* software package (v.7.0); the free energy of binding (Δ*G*) and entropy change (Δ*S*) were then calculated from the fitted values. For each experiment, at least two independent titrations were performed which were analysed independently. The resulting thermodynamic values were then averaged.

### 2.4. Crystallization of the Eg5–ispinesib complex   

Purified Eg5 at 10 mg ml^−1^ was mixed with 1 m*M* Mg^2+^ATP and then incubated with ispinesib at a final concentration of 1 m*M* for 2 h at 277 K; the sample was then centrifuged at 14 000*g* for 5 min at 277 K to pellet undissolved inhibitor. Initial crystals of the complex were obtained at 277 K by vapour diffusion in sitting or hanging drops consisting of 200 nl protein–inhibitor complex and 200 nl reservoir solution equilibrated against a reservoir consisting of 0.1 *M* Tris pH 8.5, 0.02 *M* MgCl_2_, 20%(*w*/*v*) polyethylene glycol (PEG) 8000. Crystals were subsequently grown using identical conditions in 24-well plates (Linbro, Hampton Research) using drops consisting of 1 µl protein solution and 1 µl reservoir solution and were improved by streak-seeding to generate crystals with a rectangular plate morphology that were suitable for data collection. Prior to data collection, crystals were immersed in cryoprotectant solution [0.12 *M* Tris pH 8.5, 0.024 *M* MgCl_2_, 24%(*w*/*v*) PEG 8000, 15% glycerol] and flash-cooled in liquid nitrogen.

### 2.5. Data collection, structure determination, refinement and model quality   

**Figure 1:**
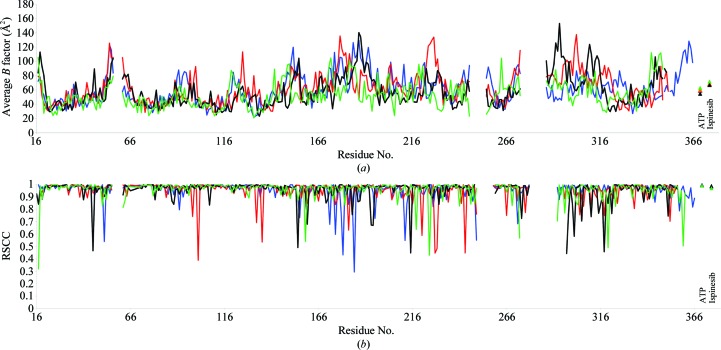
Plots of per-residue average *B* factors (*a*) and real-space correlation coefficients (*b*) for chains *A* (blue), *B* (red), *C* (black) and *D* (green). The real-space correlation coefficient (RSCC) was calculated with *SFCHECK* (Vaguine *et al.*, 1999 ▶) using a σ_A_-weighted 2*F*_o_ − *F*_c_ map.

Diffraction data were collected at 100 K on beamline I02 at Diamond Light Source. Data were processed and scaled to 2.6 Å resolution using *XDS*/*XSCALE* (Kabsch, 2010 ▶), then truncated and further processed with the *CCP*4 suite of programs (Winn *et al.*, 2011 ▶). The structure of the Eg5–ispinesib complex was solved in space group *P*2_1_ by molecular replacement with *MOLREP* using the Eg5 tetramer of PDB entry 2gm1 as the search model (Kim *et al.*, 2006 ▶). Twinning analysis was carried out with *phenix.xtriage* from the *PHENIX* suite (Adams *et al.*, 2002 ▶). Iterative improvement of this structure then proceeded through cycles of model building with *Coot* (Emsley & Cowtan, 2004 ▶) and refinement using *PHENIX* or *REFMAC*5 (Murshudov *et al.*, 2011 ▶), resulting in a final model with an *R*_free_ of 25.3% and overall reasonable geometry. Coordinates and dictionaries for ispinesib were obtained from the Dundee *PRODRG* server (Schüttelkopf & van Aalten, 2004 ▶). Crystallographic statistics are given in Table 1 ▶. Co­ordinates and structure factors have been deposited in the Worldwide Protein Data Bank (PDB entry 4ap0). In the Ramachandran plot, 98.1% of the residues are in preferred regions, 1.9% of the residues are in allowed regions and there are no outliers (as calculated by *MolProbity*; Chen *et al.*, 2010 ▶). Plots of per-residue real-space correlation coefficients (calculated with *SFCHECK*; Vaguine *et al.*, 1999 ▶) and *B* factors are shown in Fig. 1 ▶.

## 3. Results and discussion   

### 3.1. Biochemical and biophysical investigation of ispinesib binding   

**Figure 2:**
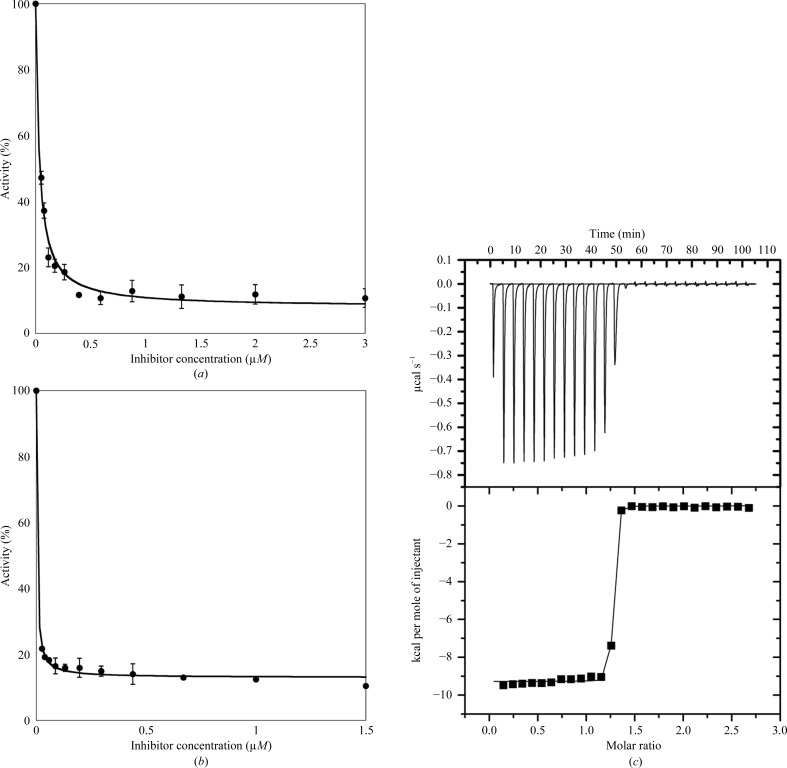
Characterization of the inhibition of Eg5 by ispinesib. Inhibition of the (*a*) basal and (*b*) MT-stimulated ATPase activity of Eg5. (*c*) Raw (top) and integrated (bottom) ITC data demonstrating saturable exothermic evolution of heat upon sequential additions of ispinesib to Eg5. 1 cal = 4.184 J.

While it has been well established that ispinesib inhibits the MT-stimulated Eg5 activity with low nanomolar affinity (IC_50_ = 5.0 ± 0.5 n*M*; Sheth *et al.*, 2009 ▶), we were interested in determining whether the same holds true in the absence of MTs in order to obtain an indication of whether Eg5–ispinesib cocrystallization (which precludes the presence of MTs) would be likely to succeed in our hands (Table 2 ▶, Figs. 2 ▶ *a* and 2 ▶ *b*). While the IC_50_ estimate obtained for MT-stimulated ATPase activity (IC_50,MT_ = 3.0 ± 0.4 n*M*) agrees well with the literature data, in the absence of MTs the IC_50_ of ispinesib increases by one order of magnitude to 32.8 ± 0.5 n*M*. Given the different protein concentrations used in the assays, it is not uncommon for tight-binding inhibitors to appear less potent against the basal compared with the MT-stimulated ATPase activity of kinesins. However, this drop in affinity should not, and indeed does not, impede complex crystallization. Sheth *et al.* (2009 ▶) also performed microcalorimetric binding studies on the Eg5–ispinesib system (now in the absence of MTs), which yielded dissociation constants of less than 10 n*M*. To further investigate this discrepancy, we repeated the ITC experiment on the binding of ispinesib to Eg5 (Table 3 ▶, Fig. 2 ▶ *c*). Our calorimetric measurements show that even in the absence of MTs ispinesib is a tight-binding Eg5 ligand (*K*_d_ < 10 n*M*), which is in agreement with the data of Sheth *et al.* (2009 ▶).

### 3.2. Overall structure   

**Figure 3:**
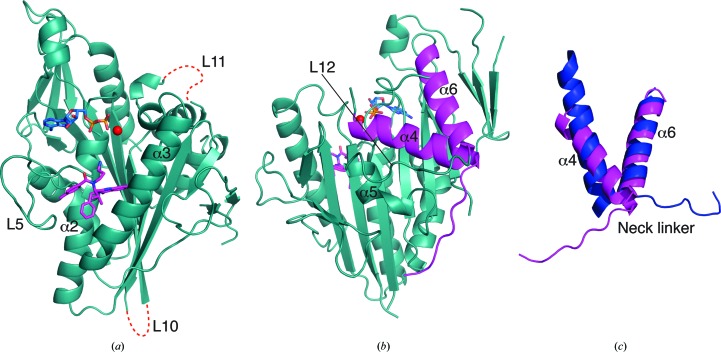
Overall structure of the ADP–Eg5–ispinesib ternary complex (chain *B*). (*a*) Front view of the Eg5 motor domain in complex with Mg^2+^ (red), ADP (blue) and ispinesib (magenta); red dotted lines indicate the locations of the disordered loops L10 and L11, and selected secondary-structure elements and loops are labelled. (*b*) Back view of the Eg5 motor domain with α4 of the switch II cluster, the neck-linker region as well as the preceding α6 helix highlighted in magenta. (*c*) Detailed view of helices α4 and α6 as well as the neck-linker region of the Eg5–ispinesib complex (magenta) superimposed on the apo Eg5 structure (blue; PDB entry 1ii6; Turner *et al.*, 2001 ▶).

The structure of the Eg5–ispinesib complex was solved at 2.6 Å resolution and refined to an *R*_free_ of 25.3% with four protein molecules in the asymmetric unit. Depending on the molecule, the N-terminal 15–17 residues as well as the C-­terminal 2–9 residues of Eg5_1–368_ are disordered, as are a number of loops; in particular, loops L10 and L11 (Fig. 3 ▶) are absent from all four molecules. Aside from this, the four independent complexes in the asymmetric unit are mostly similar, with pairwise superpositions giving r.m.s. deviations of around 0.6 Å for ∼300 C^α^ atoms. For the sake of clarity, the further discussion will thus focus only on chain *B* unless stated otherwise.

The present structure conforms to the canonical kinesin motor domain fold with an eight-stranded β-sheet sandwiched between three major α-helices on either side (Figs. 3 ▶ *a* and 3 ▶ *b*). It shows one molecule of Mg^2+^ADP bound in the nucleotide-binding pocket with the magnesium coordinated by the β-­phosphate, the side-chain hydroxyl of Thr112 and three water molecules, resulting in an octahedral geometry with one disordered ligand (presumably bulk solvent). Ispinesib occupies the inhibitor-binding pocket formed by helix α2, loop L5 and helix α3 (Fig. 3 ▶ *a*).

Comparison with apo Eg5 (PDB entry 1ii6; Turner *et al.*, 2001 ▶) shows that the region around the inhibitor-binding pocket undergoes major conformational changes on ispinesib binding. Additional changes extend towards the other end of the motor domain, bringing about larger conformational changes in the switch II cluster (helix α4, loop L12 and helix α5) and the neck-linker region (Yan *et al.*, 2004 ▶). All four molecules in the asymmetric unit depict the final ispinesib-bound state. Helix α4 rotates and moves by around 7 Å in the inhibitor-bound state compared with the native structure. The anticlockwise rotation and shift of helix α4 rearranges the switch II cluster and opens up space enabling the neck-linker to dock to the motor domain (Figs. 3 ▶ *b* and 3 ▶ *c*). This shows that ispinesib brings about structural changes in the Eg5 catalytic domain, in agreement with published biochemical data (Lad *et al.*, 2008 ▶).

### 3.3. Ispinesib binding to Eg5 and comparison with other allosteric inhibitors   

**Figure 4:**
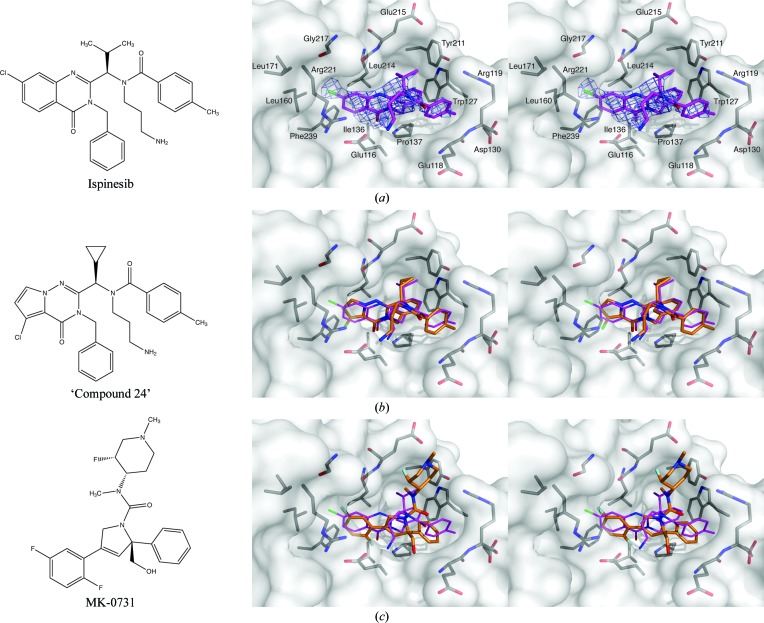
Interactions of ispinesib with the Eg5 inhibitor-binding region and comparison with other inhibitors of Eg5. (*a*) Chemical structure of ispinesib (left); stereoview of ispinesib (purple sticks) bound to the allosteric site of Eg5. Side chains and/or backbone atoms of interacting protein residues are shown as grey sticks and labelled; the protein surface is displayed semitransparently. Unbiased (*i.e.* calculated prior to including the ligand in the model) σ_A_-­weighted *F*_o_ − *F*_c_ electron density for the ligand contoured at 2.5σ is shown in slate. (*b*) Chemical structure (left) and comparison of the Eg5 binding mode (right) of ‘compound 24’ (orange). (*c*) Chemical structure (left) and comparison of the Eg5 binding mode (right) of MK-0731 (orange).

Ispinesib is buried in the allosteric site and displays numerous interactions with residues of the inhibitor-binding pocket (Fig. 4 ▶ *a*; Zhang *et al.*, 2008 ▶). At the resolution obtained for this structure, we did not observe any ordered water molecules in close proximity to the inhibitor. The benzyl moiety of the ligand is buried deeply in the hydrophobic part of the pocket, where it stacks with the Pro137 ring and makes an edge-to-face interaction with the side chain of Trp127, as well as hydrophobic interactions with the side chains of Tyr211 and Leu214. In addition, the benzyl group also forms an intramolecular edge-to-face stacking interaction with the *p*-­toluyl moiety of the inhibitor, which in turn stacks extensively with the mostly flat protein backbone of Glu118/Arg119 and also interacts with parts of the side chains of Arg119, Trp127 and Asp130. The isopropyl group of ispinesib is only partly buried between the side chains of Tyr211 and Leu214 as well as the backbone of the latter residue and Glu215. The chlorine substituent of the 7-chloro-3,4-dihydro-4-oxo-3-­(phenylmethyl)-2-quinazolinyl moiety sits in another mostly hydrophobic pocket formed by the backbone of Gly217 and the side chains of Leu160, Leu171 and Arg221. In three of the four chains (*B*/*C*/*D*) the primary amine of ispinesib is oriented towards the ADP-binding site, and while it is mostly solvent-exposed the amine can interact favourably with the anionic Glu116 side chain. In chain *A*, in contrast, the aminopropyl moiety is disordered. Taken together, the intrinsic flexibility of the aminopropyl group, together with the observed conformational variability and its mostly solvent-exposed position, suggests that this functional group is less important for the binding of ispinesib to Eg5 than in other Eg5-targeting compounds which also contain this primary amine. This is further underlined by STLC and related analogues: in this case the primary amine is absolutely essential for Eg5 inhibition (Debonis *et al.*, 2008 ▶) and an analogue with a tertiary amine completely abolishes inhibition, whereas a tertiary amine in ispinesib is still capable of inhibiting Eg5 in the low-nanomolar range (Sakowicz *et al.*, 2004 ▶). It is remarkable that aside from the salt bridge between this primary amine and the Glu116 carboxylate, ispinesib makes exclusively hydrophobic interactions with the protein. In this context, it is particularly noteworthy that the carbonyl oxygen group of the quinazolin-4-one ring system is buried in a hydrophobic pocket, which frustrates its hydrogen-bonding potential. While several potential weak C—H⋯O hydrogen bonds are likely to at least partially compensate for this, the replacement of this group by a similarly sized hydrophobic group should provide improvements to Eg5 binding, although these modifications will have to be carefully balanced with its drug-like properties.

Although the unavailability of coordinates/structure factors precludes a detailed analysis, we felt it would be informative to compare our structure with the previously published Eg5–ispinesib complex (Zhang *et al.*, 2008 ▶). Overall, the two models adopt a similar ‘final state’ conformation, although the previous structure exhibits a number of features that are unusual among published Eg5 structures and are not replicated by our model. Specifically, the helix between β1 and β1a, as well as parts of the central β-sheet around β6/β7, are shifted significantly with respect to the present complex structure. Additionally, loop 11 is ordered in the former structure, while in essentially all other Eg5 complex structures it is disordered. Zhang and coworkers suggest that these differences from the ‘canonical’ Eg5 conformation are a consequence of crystal contacts, which is plausible given their unique unit-cell parameters and is furthermore compatible with these features being absent from the present structure. Based on the discussion and figures provided by Zhang and coworkers, the conformation and binding mode of ispinesib appears to be virtually identical in the two structures, down to the orientation of the flexible aminopropyl group. While the isopropyl moiety of the ligand was apparently and unexpectedly modelled as a flat group in the previous structure, this has little effect on the observed ligand–protein interactions.

To further investigate the binding mode of ispinesib, we compared the structure of ispinesib-bound Eg5 with the Eg5-complex structures of two other related inhibitors: the pyrrolotriazin-4-one-based ‘compound 24’ (PDB entry 2gm1; Kim *et al.*, 2006 ▶) and MK-0731 (PDB entry 2cjo; Cox *et al.*, 2008 ▶) (Figs. 4 ▶ *b* and 4 ▶ *c*).

Aside from the differences in the core ring system, ‘compound 24’ is virtually identical to ispinesib and it is thus not surprising that the two molecules adopt similar binding modes, with the benzyl, *p*-toluyl and aminopropyl groups all adopting consistent conformations and making equivalent interactions with the protein (Fig. 4 ▶ *b*). The cyclopropyl group of compound 24 takes the place of the isopropyl group of ispinesib and again makes equivalent interactions. The most significant difference between these two compounds lies in the different attachment of the chlorine substituent to the core ring system, which is partly necessitated by the change from a quinazolinone (ispinesib) to a pyrrolotriazinone (compound 24). While the chlorine of ispinesib makes its closest contacts with the Leu160 side chain and the backbone of Gly217, the chlorine of compound 24 points deeper into the binding pocket (the angle between the two C—Cl bonds after superposition is ∼104°), where it interacts predominantly with the side chains of Ile136 and Phe239. Given that the almost perfect alignment of the two core ring systems after superposition of the two complex structures on the protein component (*cf.* Fig. 4 ▶ *b*) supports a lack of excessive strain owing to the presence of either chlorine, thus might suggest that the Eg5 affinity of either compound could be improved by introducing additional substitutions on the benzo and pyrrolo ring, respectively. At the same time, compound 24 is almost two orders of magnitude less potent as an Eg5 inhibitor compared with ispinesib (Kim *et al.*, 2006 ▶), which might mean that it is compound 24 rather than ispinesib that would benefit most from such reciprocal elaboration.

While at first glance the 3-phenyl-dihydropyrrole-based MK-0731 shares few chemical features with ispinesib, a superposition of the two Eg5 complexes reveals the presence of several congruent structural elements (Fig. 4 ▶ *c*). The difluoro­phenyl ring of MK-0731 takes the place of the quinazolinone system of ispinesib, with one fluorine mimicking the chlorine substituent of ispinesib and the second fluorine superimposing reasonably well with the quinazol­one oxo group, although owing to the smaller size of F compared with Cl as well as the steric requirements of the rest of the molecule MK-0731 inserts less deeply into the binding pocket than does ispinesib. The unsubstituted phenyl ring of MK-0731 binds in the general area occupied by the chemically similar benzyl and *p*-­toluyl groups of ispinesib. The reduced bulk and the positioning of this phenyl enable the side chain of Arg119 to close as a lid over this part of MK-0731, enabling favourable stacking interactions that are not accessible to ispinesib. A feature unique to MK-0731 is the hydroxymethyl group attached to the central dihydropyrrole ring. Introduced to increase polarity and thus reduce hERG binding (Cox *et al.*, 2008 ▶), this group can be considered as related in purpose to the aminopropyl moiety of ispinesib, but its reduced intrinsic flexibility combined with local steric hindrance makes the hydroxymethyl group a potentially better choice that may inspire similar modifications in future ispinesib derivatives, although they will have to be monitored for possible phase II metabolic liabilities. Another feature unique to MK-0731 is its fluoro­methylpiperidine ‘side chain’. While it does interact with the protein, this moiety is mostly solvent-exposed and as such enables modulation of the physicochemical properties and thus the pharmacokinetics of the inhibitor. Despite the divergent binding features, ispinesib and MK-0731 are remarkably similar in their affinity for Eg5 (the IC_50_ of MK-­0731 for MT-stimulated Eg5 is 2 n*M*; Cox *et al.*, 2008 ▶), again suggesting that ‘transplanting’ features may yield improved Eg5 ligands.

### 3.4. Twinning and pseudotranslational symmetry   

Several data sets were collected from Eg5–ADP–ispinesib complex crystals, most of which could not be processed with any commonly used software. The present data could be indexed, processed and scaled in both primitive orthorhombic and *C*-centred monoclinic space groups. Structure solution by molecular replacement in these space groups was possible, yielding initially reasonable models, all of which subsequently failed to refine either because the *R* values could not be decreased or because model completion revealed unavoidable clashes or otherwise impossible molecular arrangements. Decreasing the lattice symmetry to primitive monoclinic allowed the structure to be solved in space group *P*2_1_ for all three possible choices of crystallographic symmetry axis. Two of the three cell choices allowed model improvement through iterative refinement, while the third was yet again unrefinable. Inspection of the symmetry relationships between protein molecules in the *P*2_1_-refined models revealed three orthogonal twofold symmetry axes, two of which can individually function as crystallo­graphic symmetry elements, while the third has a screw component of ∼0.38 and as such provides only noncrystallographic symmetry. These findings explain the lack of refinement of the various orthorhombic models as well as one of the three *P*2_1_ axis choices, although it is somewhat surprising that believable models could be obtained from molecular replacement in these cases at all, as interpreting the ‘odd’ screw axis as a crystallographic 2_1_ symmetry axis would impose a coordinate shift of more than 13 Å on parts of the model.

In addition, the *P*2_1_ model(s) exhibits pure translational (noncrystallographic) symmetry that almost, but not quite, mimics a face-centring operation, explaining why scaling and molecular replacement were successful in *C*2 but again did not yield a refinable model. To add insult to injury, the Eg5–ispinesib complex crystals appear to suffer from pseudo-merohedral twinning, as suggested by the Britton plot (Britton, 1972 ▶), *H*-test (Yeates, 1997 ▶) and RvR plot (Lebedev *et al.*, 2006 ▶), and supported by a significant drop in *R*/*R*_free_ of over 7% when switching from untwinned to twinned refinement in *PHENIX*, with the twin law a twofold axis orthogonal to the crystallographic symmetry axis and a twin fraction of around 0.4.

Both translational noncrystallographic symmetry and twinning complicate structure solution and even for the valid cell choices similar refinable but non-equivalent models were obtained from molecular replacement. The final structure presented here was selected based on refinement statistics, crystallographic packing and precedent for this cell choice and packing in the PDB in entry 2gm1 (Kim *et al.*, 2006 ▶), the protein component of which was subsequently used as a search model for molecular replacement.

## 4. Conclusion and biological significance   

Eg5 shows significant potential as a drug target for cancer chemotherapy and correspondingly has attracted widespread attention, with numerous inhibitors in various phases of drug development. All Eg5 inhibitors developed to date target the globular motor domain, where they bind to one of two distinct sites: ATP-competitive inhibitors bind either in or close to the ATP-binding site (P-loop; Luo *et al.*, 2007 ▶; Parrish *et al.*, 2007 ▶), whereas allosteric inhibitors bind to the L5 loop region. The allosteric Eg5 inhibitors are attractive not only because they avoid binding in competition with ATP/ADP, but more importantly because binding to the particularly long L5 loop of Eg5 provides these compounds with specificity over other closely related kinesins, which generally possess a much shorter L5 loop that cannot furnish a comparable binding pocket. Ispinesib is a promising allosteric Eg5 inhibitor that is currently in phase II clinical trials. It is thus surprising that no structural data for the Eg5–ispinesib complex have been available to date. The present structure of the ternary Eg5–ADP–ispinesib complex seeks to remedy this. It shows that the ligand makes extensive hydrophobic, but essentially no hydrophilic, interactions with the allosteric binding pocket of Eg5. Analysis of the binding mode, as well as a comparison with other allosteric Eg5 inhibitors, suggests a number of ways in which ispinesib could be modified while either retaining or even improving its affinity for Eg5, an argument that is further supported by the development of the second-generation ispinesib-based analogue SB-743921 (Holen *et al.*, 2011 ▶) and the amount of additional data on development of ispinesib-related compounds in the patent literature (Matsuno *et al.*, 2008 ▶). This information should prove invaluable for future iterations of this inhibitor scaffold, be it to improve potency, to alter the pharmacokinetics or to counter the ever-present threat of resistance (Jackson, 2005 ▶).

## Supplementary Material

PDB reference: Eg5–ADP–ispinesib complex, 4ap0

## Figures and Tables

**Table 1:** Data-collection and structure-refinement statistics for the Eg5ispinesib complex Values in parentheses pertain to the highest resolution shell of 0.15. Ramachandran plot statistics were obtained with *MolProbity* (Chen *et al.*, 2010 ▶).

Unit-cell parameters (, )	*a* = 64.7, *b* = 112.6, *c* = 106.9, = 90.0
Space group	*P*2_1_
Molecules per asymmetric unit	4
Resolution range ()	30.02.6
Total reflections	134457
Unique reflections	45511
Completeness (%)	95.7 (91.0)
Multiplicity	3.0 (2.7)
*R*_merge_ (%)	6.9 (62.2)
*I*/(*I*)	13.3 (2.0)
*R*_work_/*R*_free_ (%)	20.4/25.3
Wilson *B* (^2^)	59.9
Average *B* (^2^)	
Overall	57.7
Protein	58.2
Solvent	48.1
ADP	33.8
Ispinesib	44.9
R.m.s.d. bond lengths ()	0.016
R.m.s.d. bond angles ()	1.47
Ramachandran plot statistics (%)	
Favoured	98.1
Allowed	1.9
Outliers	0

**Table 2:** Inhibition of Eg5 ATPase activity by ispinesib

	Basal	MT-stimulated
IC_50_ (n*M*)	32.8 0.5	3.0 0.4

**Table 3:** Thermodynamic data for the binding of ispinesib to Eg5ADP Owing to the tight binding of ispinesib, the measured apparent *K*_d_ can only be treated as an upper limit. 1cal = 4.186J.

*n*	*K*_d_^app^ (n*M*)	*H* (kcalmol^1^)	*T* *S* ^app^ (kcalmol^1^)
1.270 0.003	10	9.08 0.04	2.81 0.23
